# Editorial: Multi-species biofilms in the food industry

**DOI:** 10.3389/fmicb.2022.1023428

**Published:** 2022-09-09

**Authors:** Lei Yuan, Huhu Wang, Wenzheng Liu, Faizan A. Sadiq, Yong Zhao

**Affiliations:** ^1^School of Food Science and Technology, Yangzhou University, Yangzhou, China; ^2^College of Food Science and Technology, Nanjing Agricultural University, Nanjing, China; ^3^School of Food and Pharmaceutical Engineering, Nanjing Normal University, Nanjing, China; ^4^Flanders Research Institute for Agriculture, Fisheries and Food, Merelbeke, Belgium; ^5^College of Food Science and Technology, Shanghai Ocean University, Shanghai, China

**Keywords:** multi-species biofilms, interspecies interactions, persistence, resistance, biofilm control strategies

Microbial biofilms are spatially organized communities of microorganisms attached to different surfaces and embedded within a self-produced matrix composed of extracellular polymeric substances (EPS). The formation of biofilms frequently occurs in the food industry, due to available nutrients, large areas for microbial growth, and the complexity of processing facilities. Microbial biofilms have represented an outstanding survival strategy against stressful conditions for both spoilage and pathogenic bacteria during food processing (Alvarez-Ordóez et al., 2019). Thus, microbial biofilms have been notoriously identified as causes for food spoilage, foodborne diseases, and high costs for equipment damage in the food industry.

Most knowledge regarding food biofilms is based on the formation and control of single-species biofilms; however, they ignored the fact that mixed-species biofilms represent the most frequent form of contamination in the food industry, and bacterial interactions may facilitate the properties and behavior quite different from single-species biofilms (Yuan et al., 2020). A better understanding of the metabolic activity of microorganisms in mixed-species biofilms is challenging, but in turn, will definitely be useful for the design of more efficient strategies for controlling unwanted biofilms in the food industry.

In this context, this Research Topic aims to collect recent studies on the following themes: (1) the mechanisms underlying the formation of mixed-species biofilms; (2) the persistence and resistance under food processing environments; (3) the development of appropriate methods to study mixed-species biofilms; and (4) the potential novel strategies to control the formation of mixed-species biofilms. This Research Topic comprises 4 original research articles from Austria, China, Canada, and South Korea, contributed by 24 authors. Two contributions focused on the evaluation of mixed-species biofilm formation, and the others designed potential effective methods to control the formation of multi-species biofilms.

Voglauer et al. described the complexity of mixed-species biofilms formed in water hoses of a meat processing environment by high-throughput 16S rRNA gene sequencing, with *Comamonadaceae* and *Pseudoxanthomonas* being most abundant in biofilms. Opportunistic pathogens, including the genera of *Neochlamydia, Legionella*, and *Pseudomonas*, have also been detected with different abundances. However, knowledge of the effects of environmental factors on the formation of biofilms in water hoses is limited. The authors also stated that control strategies have to be taken to prevent the colonization and biofilm formation in water hoses in order to assure water safety for the health of workers and consumers. In another study, Nan et al. evaluated the formation and transfer of mixed-species biofilms by *Escherichia coli* O103:H2 and lactic acid bacteria or spoilage bacteria at different temperatures, storage time, and humidity conditions. They stated that the formation of mixed-species biofilms may increase or diminish the risk of beef contamination by *E. coli* O103:H2, depending on the bacteria community and environmental conditions in beef processing environment.

It is generally believed that mixed-species biofilms exhibit higher resistance to biofilm control strategies in food processing facilities when compared to single-species biofilms (Pang et al., 2017; Pang and Yuk, 2018). This emphasizes the importance for the development of effective biofilm control methods. In this Research Topic, two research articles are related to the control of mixed-species biofilms formed by pathogenic or spoilage microorganisms in the food industry. Yan et al. confirmed the high antibiofilm efficiency of lightly acidic electrolyzed water by preventing surface adhesions and impairing biofilm cell membrane integrities, and the antimicrobial activity highly depends on its storage time. This study also investigated the inhibitory effect of slightly acidic electrolyzed water on mixed-species of *Listeria monocytogenes* Scott A and *Staphylococcus aureus* for the first time.

Hurdle technology, the combination of two or more control strategies, has been proved as a promising technology to effectively remove biofilm cells from food processing facilities, as they would attack microorganisms in different ways (Yuan et al., 2021). In another contribution, Zhu et al. examined the synergistic effect of ε-Polylysine hydrochloride and cinnamon essential oil against dual-species biofilms by *L. monocytogenes* and *Pseudomonas lundensis* in meat processing. The combination of the two preservatives exhibited increased inhibitory effect against dual-species biofilm formation according to the thin spare spatial structures and reduced AI-2 activity, and dramatically eradicated the preformed dual-species biofilms.

The above studies have expanded our understanding on this topic; however, relevant studies about (1) the mechanisms underlying the formation of multi-species biofilms by omics, and (2) the development of appropriate methods to study multi-species biofilms and still needed for a better understanding of multi-species biofilms.

## Author contributions

All authors listed have made a substantial, direct, and intellectual contribution to the work and approved it for publication.

## Funding

This research was financially supported by the Natural Science Foundation of Jiangsu Province (BK20210814) and China Postdoctoral Science Foundation (2021TQ0274).

## Conflict of interest

The authors declare that the research was conducted in the absence of any commercial or financial relationships that could be construed as a potential conflict of interest.

## Publisher's note

All claims expressed in this article are solely those of the authors and do not necessarily represent those of their affiliated organizations, or those of the publisher, the editors and the reviewers. Any product that may be evaluated in this article, or claim that may be made by its manufacturer, is not guaranteed or endorsed by the publisher.
